# Preservation of Beef with Limonene-Rich Citrus Peel Extracts: Antioxidant, Antimicrobial and Textural Benefits

**DOI:** 10.3390/foods14203506

**Published:** 2025-10-15

**Authors:** Chunlong Liu, Shiyang Xu, Xiuping Liu, Wenxia Wang, Wenzhen Liao, Xingfen Yang, Qi He

**Affiliations:** 1 Food Safety and Health Research Center, Guangdong Provincial Key Laboratory of Tropical Disease Research, BSL-3 Laboratory (Guangdong), School of Public Health, Southern Medical University, Guangzhou 510640, China; lianyue886@163.com (C.L.); 202332615@mail.sdu.edu.com (S.X.); liuxiuping0625@163.com (X.L.); wenzhenliao@smu.edu.cn (W.L.); 2 State Key Laboratory of Microbial Technology, Shandong University, Qingdao 266237, China; 3 School of Biomedical and Pharmaceutical Sciences, Guangdong University of Technology, Guangzhou 510006, China; fewwxia@gdut.edu.cn; 4 Institute of Microbiology, Chinese Academy of Sciences, Beijing 100101, China

**Keywords:** citrus peel beef, sensory decline, lipid oxidation, protein degradation, microstructure maintain, biogenic amines accumulation

## Abstract

Citrus peels, long used in traditional food preservation, are rich in bioactive compounds with potential antioxidant and antimicrobial properties. However, a systematic comparison of the efficacy of different citrus varieties and the underlying mechanisms in meat preservation remains limited. This study investigated the chemical composition of peels from four citrus varieties (*Citrus reticulata*, CR; *C. sinensis*, CS; *C. bigarradia*, CB; and *C. macrocarpa*, CM) and their efficacy in preserving beef quality during refrigerated storage. GC-MS analysis revealed limonene as the predominant volatile component (59.6~77.1%), with CR peel exhibiting the highest content (77.1%). CR extract also demonstrated superior antioxidant activity (DPPH: 60.8%; ABTS: 66.0%) and antimicrobial effects against five common meat microbial species. Beef samples treated with CR peel extract significantly (*p* < 0.05) reduced lipid oxidation (TBARS: 2.88 vs. 4.83 mg MDA/kg in control) and protein degradation (TVB-N: 270 vs. 371 mg/kg). Microstructural integrity was better maintained, as evidenced by lower surface hydrophobicity, higher sulfhydryl content, and reduced carbonyl formation. Furthermore, CR treatment suppressed microbial growth (TBC and TAC reduced by ~30%) and the accumulation of spoilage-related biogenic amines, particularly putrescine (12~18.8 vs. 27.4 mg/kg). Correlation analysis identified limonene content as strongly correlated with antioxidant and antimicrobial activities. This work validates the scientific basis of using citrus peel, particularly CR, as a natural preservative, effectively bridging traditional culinary practice with modern food science by elucidating its multi-target role in extending the shelf life and enhancing the safety of beef.

## 1. Introduction

Citrus peels, representing a significant portion of citrus processing waste, are increasingly recognized not as mere byproducts but as valuable reservoirs of bioactive compounds [1,2]. These compounds, including flavonoids (e.g., hesperidin, naringin, and polymethoxyflavones), phenolic acids, and alkaloids, have been extensively documented for their broad-spectrum biological activities [3,4]. Notably, their potent antioxidant and antimicrobial properties underpin a considerable potential for application in food preservation systems, offering a natural alternative to synthetic additives [5]. The utility of these peels in inhibiting lipid oxidation—a primary cause of quality deterioration in meat products—and in suppressing the growth of common spoilage microorganisms presents a promising avenue for enhancing food safety and extending shelf life [6,7].

Despite the well-documented bioactivity of citrus peel constituents, a significant research gap persists in their systematic application to meat preservation, particularly concerning the impact of varietal differences. The chemical composition and, consequently, the functional efficacy of Citrus peels are highly variable, influenced by factors such as genetic species, cultivation environment, and post-harvest processing [8,9]. For instance, the specific profiles of flavonoids and volatile oils can differ markedly between common varieties like tangerines (Citrus reticulata) and sweet oranges (Citrus sinensis), potentially leading to divergent preservative outcomes [10]. Previous studies have often treated “citrus peel” as a generic entity, lacking a comparative analysis of how these intrinsic variations affect their performance in complex food matrices like meat. This oversight limits the ability to select or optimize peel sources for maximal preservative effect [3].

Furthermore, while historical and traditional practices, such as “Citrus peel beef”, a traditional Chinese dish, provide compelling anecdotal evidence of its preservative role, the underlying scientific mechanisms remain inadequately explored. Most contemporary research has focused on the in vitro efficacy of crude extracts or their direct application to meat surfaces, monitoring general quality parameters like microbial counts and thiobarbituric acid reactive substances (TBARS) [11,12]. However, a more profound, mechanistic understanding is lacking. Key questions regarding the transformation of bioactive compounds (e.g., glycoside hydrolysis, oxidation) during marination and thermal processing, their interaction with meat proteins and lipids, and their specific role in mitigating quality degradation during storage are largely unanswered [13]. This lack of depth hinders the rational development and standardization of citrus peel as a natural preservative [14].

This study was therefore designed to bridge these critical knowledge gaps. We hypothesize that different citrus varieties impart distinct preservative effects on beef due to their unique phytochemical profiles and the chemical transformations these compounds undergo during processing. The objectives of this research are twofold: first, to comprehensively characterize and compare the key bioactive compounds in peels from selected citrus varieties; and second, to elucidate the transformation pathways of these compounds during the preparation and storage of citrus peel-marinated beef, correlating these changes with measurable quality indicators. By applying advanced chromatographic and spectroscopic techniques to a model traditional practice, this work seeks to move beyond phenomenological observation to provide a mechanistic foundation for the application of Citrus peels, ultimately contributing to the development of effective, natural strategies for meat preservation.

## 2. Materials and Methods

### 2.1. Materials

A total of 400 fruits (100 per species) from 4 tangerine species, namely *Citrus reticulata* (CR), *Citrus sinensis* (CS), *Citrus bigarradia* (CB), and *Citrus macrocarpa* (CM) were manually harvested from orchards located in Xinhui District (~22° N, ~113° E, ~21 °C average annual temperature, ~1700 mm annual rainfall), Jiangmen City, Guangdong Province, China, in October 2023. Fruits without damage and contamination were selected and carefully hand-peeled within 3 h of harvest. The resulting peels were thoroughly washed. To replicate the key thermal and dehydration effects of traditional sun-drying and aging processes for citrus peel [10], while ensuring batch-to-batch consistency and efficiency, drying was performed using a dryer (model 6CHZ-9B, Fujian Jiayou Co., Fuzhou, China) at 100 °C for 90 min. This protocol was established in preliminary studies as a necessary step to achieve the low moisture content (<1%) required for shelf-stability and subsequent grinding, and its impact on the specific bioactive compounds of interest is accounted for in our comparative analysis.

Fresh beef tenderloin (Longissimus dorsi muscle, 200 individuals, ~250 g/individual), sourced from a single production batch to ensure consistency, was obtained from castrated cattle (two years old, weighing 500~600 kg) through standard beef products from Kerchin Company (Tongliao, China), which adheres to international technical standards for feeding, slaughtering, processing, packaging, and transportation [15]. All samples were derived from the same anatomical location to minimize biological variation, and transported to the laboratory via a standard cold chain at approximately 1 °C within 24 h after slaughter.

All reagents used in the study were purchased from Macklin Biochemical Technology Co., Ltd. (Shanghai), Chemical Reagent Co., Ltd. (Guangzhou), and Damao Biochemical Technology Co., Ltd. (Tianjin), China.

### 2.2. Analysis of the Composition and Activities of Citrus Peel

The Citrus peels were extracted by hydrodistillation at ~100 °C for 6 h using a Clevenger-type apparatus (Kesijia Co., Beijing, China), for subsequent analysis. The composition of the Citrus peel extract was analyzed using gas chromatography-mass spectrometry (GC-MS) system (6890-5975, Agilent Technologies Co., Palo Alto, CA, USA). The separation was performed on an HP-5MS capillary column (30 m × 0.25 mm, 0.25 μm; Agilent Technologies Co.), with the oven temperature programmed as follows: initial hold at 50 °C for 2 min, ramped at 5 °C/min to 250 °C, and held for 10 min. Electron impact (EI) ionization was employed at 70 eV [16].

The antioxidant activity of Citrus peel extract was evaluated by measuring its radical scavenging capacity using two assays: 2,2-diphenyl-1-picrylhydrazyl (DPPH) and 2,2-azinobis (3-ethylbenzothiazoline-6-sulfonic) acid radical cation (ABTS^+^) [17].

The antimicrobial activity of the citrus peel extracts was evaluated against a panel of microorganisms relevant to meat spoilage and safety. The selected strains included both Gram-positive (*Staphylococcus aureus* ATCC 6538, *Listeria monocytogenes* ATCC 19115) and Gram-negative (*Pseudomonas aeruginosa* ATCC 9027, *Salmonella typhimurium* ATCC 14028, *Escherichia coli* ATCC 25922) bacteria. The antimicrobial activity was first screened by disk diffusion assay on Mueller-Hinton agar, with plates incubated at 37 °C for 18~24 h [18]. For quantitative assessment, the minimum inhibitory concentration (MIC) was determined via broth microdilution following CLSI guidelines. Briefly, each extract was serially diluted two-fold in Mueller-Hinton broth within 96-well microplates, yielding final concentrations from 0.0625 to 32 mg/mL. Each well was inoculated with approximately 5 × 10^5^ CFU/mL of bacterial suspension, prepared by adjusting to a 0.5 McFarland standard. Growth control (inoculum without extract) and sterility control (broth only) wells were included. After incubation at 37 °C for 24 h, the MIC was recorded as the lowest concentration showing no visible growth [19].

### 2.3. Processing and Storage of Citrus Peel Beef

Beef portions (More than 200 individuals, ~250 g per portion) cut into strips (1 cm × 1 cm × 3 cm) and randomly divided into 5 groups, including control group (C), and treatment groups T1, T2, T3, and T4, which corresponded to marination with extracts from *Citrus reticulata* (CR), *Citrus sinensis* (CS), *Citrus bigarradia* (CB), and *Citrus macrocarpa* (CM), respectively. Among them, the beef strips in the control group were boiled in boiling deionized water for 2 min, while the beef strips in the T1~T4 groups were boiled for 2 min in a solution containing 50 g/L of the corresponding Citrus peel powder. In all groups, the beef-to-liquid ratio was maintained at 1:10 (*w*/*v*). Subsequently, all samples were stored in freezers (BCD-405WBPZU1, Haier Co., Qingdao, China) at 2 ± 0.1 °C for 8 days. This storage duration was determined based on preliminary experiments, which indicated that control samples began to exhibit significant quality deterioration around this time point. Quality analysis was conducted at 2-day intervals, with randomly selected samples from each group analyzed at each time point (days 0, 2, 4, 6, and 8).

### 2.4. Sensory Variations in the Preserved Beef

Sensory involving color, odor, texture and general acceptability of the preserved beef were evaluated by an expert review panel consisted of 20 trained panelists (25~50 years old). Ten random beef strips from each group were anonymously presented and evaluated by a 5-point scale (0, awful extremely to 5, fresh extremely). The study adhered to ethical guidelines for protecting participants’ rights and privacy, including no coercion to participate, full disclosure of study requirements and risks, written or verbal consent of participants, no release of participant data without their knowledge, ability to withdraw from the study at any time, etc., and approved by the ethical review boards of Southern Medical University (Corresponding author’s institution), in compliance with national laws [20].

### 2.5. Hardiness and Springiness of the Preserved Beef

Hardiness and springiness of the preserved beef were determined by texture profile analysis (TPA) using a texture analyzer (Brookfield-CT3, Brookfield Co., Middleboro, MA, USA) [21]. Prior to analysis, samples were equilibrated to room temperature (20 ± 1 °C) for ~15 min. Each sample was compressed twice into the depth of 5 mm using a 6 mm cylindrical probe at a speed of 5 mm/s.

### 2.6. Bacterial Activity in the Preserved Beef

Total bacterial count (TBC) and total aerobic count (TAC) of the preserved beef were investigated to indicate the antimicrobial properties. TBC was determined using iron agar with 10 g/L NaCl, while TAC used plate count agar. Serial dilutions of beef homogenates were spread-plated and incubated at 37 °C for 24 h, following established protocols [22].

### 2.7. Protein Degradation in the Preserved Beef

Thiobarbituric acid reactive substances (TBARS) of the preserved beef were expressed by malonaldehyde equivalents [23]. Briefly, minced beef was mixed with 2-thiobarbituric acid and n-butanol with a water bath at 95 °C for 30 min. TBARS was obtained by an ultraviolet spectrophotometer (UV-1800, Shimadzu Co., Kyoto, Japan) using the following formula:

(1) $$TBARS\;(mg\;MDA/kg)\;=\;\frac{A_{532}\times \;9.48\;}{W}$$

where *A*_532_ represents absorbance at 532 nm; *W* represents sample weight (g).

Total volatile basic nitrogen (TVB-N) was determined using the semi-micro Kjeldahl method [24]. Specifically, minced beef samples were mixed with distilled water, alkalinized with MgO suspension (100 g/L), and distilled using a Kjeltec apparatus (KDY-9820, Ruibangxingye Co., Beijing, China). TVB-N content was calculated from the volume of hydrochloric acid required to neutralize the distilled basic nitrogen compounds.

### 2.8. Microstructure Integrity in the Preserved Beef

Surface hydrophobicity was quantified following the bromophenol blue (BPB) binding method [25]. Briefly, 0.5 g minced beef sample was homogenized in 10 mL distilled water. Subsequently, 1.0 mL of BPB solution (1.0 mg/mL) was added. After vortexing and incubation (10 min, 25 °C), the mixture was centrifuged (4000× *g*, 15 min, 4 °C). The absorbance of the supernatant was measured at 595 nm. Surface hydrophobicity (expressed as μg BPB per mg protein) was calculated based on the decrease in supernatant absorbance relative to a BPB reference standard.

Total sulfhydryl groups were determined using Ellman’s reagent (5,5′-dithiobis (2-nitrobenzoic acid), DTNB [26]. A 0.5 g minced beef sample was homogenized in 5.0 mL of phosphate-buffered saline (PBS: 10 mmol/L phosphate, 0.1 mol/L NaCl, pH 7.4). The homogenate (0.5 mL) was mixed with 4.5 mL of Tris-HCl buffer (0.2 mol/L, pH 8.2, containing 10 mmol/L EDTA). Then, 0.1 mL of DTNB solution (10 mmol/L in Tris-HCl buffer) was added. After incubation in the dark (40 °C, 25 min), the absorbance was measured at 412 nm. Total sulfhydryl content was calculated using the molar extinction coefficient of 13,600 M^−1^ cm^−1^ and expressed as nmol SH per mg protein.

Carbonyl content was assessed according to the DNPH derivatization method [27]. A 0.5 g minced beef sample was homogenized in 5.0 mL of PBS (10 mmol/L, pH 6.5). The homogenate was divided into two aliquots (1 mL each). One aliquot was treated with 1.0 mL of 10 mmol/L 2,4-dinitrophenylhydrazine (DNPH) in 2 mol/L HCl (sample). The other aliquot was treated with 1.0 mL of 2 mol/L HCl alone (blank). Both were incubated in the dark (1 h, 25 °C) with vortexing every 15 min. Proteins were precipitated by adding 1.0 mL of 20% *w*/*v* trichloroacetic acid (TCA) and centrifuged (8000× *g*, 5 min, 4 °C). The pellets were washed three times with 1 mL ethanol–ethyl acetate (1:1, *v*/*v*) to remove excess DNPH. The final pellet was dissolved in 1.5 mL of 6 mol/L guanidine hydrochloride (pH 6.5) by incubating at 37 °C for 15 min with vortexing. Absorbance was measured at 370 nm. Carbonyl content was calculated using the molar extinction coefficient of 22,000 M^−1^ cm^−1^ and expressed as nmol carbonyl per mg protein.

### 2.9. Biogenic Amines Accumulation in the Preserved Beef

The 7 main biogenic amines in the preserved beef, including histamine, putrescine, cadaverine, spermine, spermidine, tyramine, and tryptamine, were analyzed [28]. Briefly, minced beef was homogenized by 0.1 mol/L hydrochloric acid and then centrifuged. The supernatants were derivatized by mixing with saturated NaHCO_3_, 2 mol/L NaOH, and 10 mg/mL dansyl chloride-acetone solution, followed by incubation in the dark at 40 °C for 45 min. The derivatized samples were analyzed using an HPLC system (LC-20AT, Shimadzu Co., Kyoto, Japan) equipped with a C18 reversed-phase column (250 mm × 4.6 mm, 5 μm; e.g., Agilent ZORBAX Eclipse Plus) and a UV/Vis detector. The separation was achieved using a mobile phase composed of ammonium acetate buffer (20 mM, pH 6.5) and acetonitrile with the following gradient program: 0~10 min, 50~70% B; 10~15 min, 70~90% B; 15~20 min, 90% B; 20~22 min, 90~50% B; followed by 5 min re-equilibration. The flow rate was 1.0 mL/min, the column temperature was maintained at 30 °C, and the detection wavelength was set at 254 nm.

### 2.10. Correlation Analysis

Pearson correlation analysis was conducted to examine the relationships among key quality attributes of preserved peppers, using Origin 2021 software (OriginLab Co., Northampton, MA, USA) [29]. The analyzed parameters were categorized as follows: (1) Major volatile components in the Citrus peels: α-Pinene, Sabinene, β-Pinene, β-Myrcene, Limonene, and γ-Terpinene. (2) Antioxidant properties of the Citrus peels: DPPH radical scavenging rate and ABTS^+^ radical scavenging rate. (3) Antibacterial properties of the Citrus peels: Inhibition zone diameters against *S. aureus*, *P. aeruginosa*, and *E. coli*. (4) Storage quality parameters of beef at the end of storage: Sensory acceptance score, texture characteristics (hardness and elasticity), microbial indicators (TBC and TAC), chemical indicators (TBARS and TVB-N), and microstructure indicators (surface hydrophobicity, sulfhydryl content, and carbonyl content). (5) Biogenic amine accumulation in beef at the end of storage: Histamine, Putrescine, Spermine, Spermidine, Tyramine, and Tryptamine.

Before correlation analysis, all data were checked for normality and homoscedasticity to ensure the validity of Pearson’s correlation coefficients. Correlation matrices were constructed to identify significant associations among all the used parameters. Statistical significance was set at *p* < 0.05.

### 2.11. Statistical Analysis

For all assays, data were collected from independent samples (*n* = 3 per group per time point). The results are expressed as mean ± standard deviation. Statistical analysis was performed using SPSS 22.0 (SPSS Inc., Chicago, IL, USA). A two-way ANOVA was applied to evaluate the effects of treatment, storage time, and their interaction. Where significant effects were found (*p* < 0.05), Tukey’s post hoc test was used for multiple comparisons. A *p*-value < 0.05 was considered statistically significant.

## 3. Results and Discussion

### 3.1. Component Extract and Characterization of Citrus Peel

As shown in Table 1, the chemical profile of citrus peel extracts observed in this study is consistent with, yet expands upon, previous scientific reports. Our finding that limonene was the predominant volatile component (59.6~77.1%) aligns with its well-documented status as a major constituent of citrus essential oils. For instance, Sanches et al. [5] reported limonene as the primary compound in several Citrus peels, though the concentration range in our study for C. reticulata (77.1%) exceeds some literature values, potentially due to varietal differences or our specific drying process. Furthermore, the significant variation in γ-terpinene content (4.7~9.5%) across the four varieties corroborates the findings of Lima et al. [9], who highlighted substantial chemotypic diversity within the Citrus genus, influenced by genetic and environmental factors. The notable abundance of α-pinene in CB peel (5.4%) is in agreement with its reported presence in certain bitter orange varieties, as characterized by Liu et al. [30]. Finally, the detection of oxygenated monoterpenes like linalool and aldehydes such as decanal is consistent with the comprehensive volatile profiles detailed by Akhavan-Mahdavi et al. [31], confirming the complex mixture of bioactive compounds present in Citrus peels that contribute to their overall functional properties.

### 3.2. Characterization of Citrus Peel Activities

The antioxidant capacity of the citrus peel extracts, as quantified by DPPH and ABTS^+^ assays (Table 2), demonstrated significant variability among the four varieties, with CR extract exhibiting the highest activity (DPPH: 60.8%; ABTS: 66.0%). This superior antioxidant performance of CR correlates well with its highest limonene content (77.1%, Table 1), a compound renowned for its radical scavenging ability [37,41]. The overall antioxidant potency observed in our extracts aligns with the range reported for various Citrus peels. For instance, Sanches et al. (2022) documented DPPH radical scavenging activities between 45% and 65% for extracts from different mandarin hybrids, placing our results for CR at the higher end of this spectrum, potentially due to varietal superiority or extraction efficiency [5]. Furthermore, the strong positive correlation between limonene content and antioxidant activity observed in our results is mechanistically supported by Akhavan-Mahdavi et al. (2022), who detailed limonene’s role in donating hydrogen atoms to stabilize free radicals and inhibit lipid peroxidation chains [31,42].

In addition to their antioxidant effects, the citrus peel extracts displayed notable, variety-dependent antimicrobial activity against a panel of foodborne and spoilage microorganisms (Table 2). The CR extract again proved most effective, exhibiting the lowest MIC (4.1~8.3 mg/mL) and the largest inhibition zones (8.6~11.6 mm) against all microbial species. The antimicrobial efficacy can be attributed to the synergistic action of its high limonene content and other minor terpenoids. Our findings are consistent with the broad-spectrum antimicrobial nature of citrus essential oils. The activity against *S. aureus* observed here is notably higher than that reported by Liu et al. (2012) for sweet orange oil components alone, suggesting a potential synergistic enhancement in our whole peel extract [30]. The mechanism likely involves the disruption of microbial membrane integrity by lipophilic compounds like limonene, leading to leakage of cellular contents, as previously elucidated by Su et al. (2020) in their study on D-limonene emulsions [37]. The variation in efficacy against Gram-negative (*P. aeruginosa*, *E. coli*, and *S. typhimurium*) versus Gram-positive (*S. aureus* and *L. monocytogenes*) bacteria underscores the influence of cell wall structure on the extract’s penetration and action, a common observation in studies of plant-derived antimicrobials [15,37].

### 3.3. Sensory and Texture Variations in the Beef

**Figure 1:**
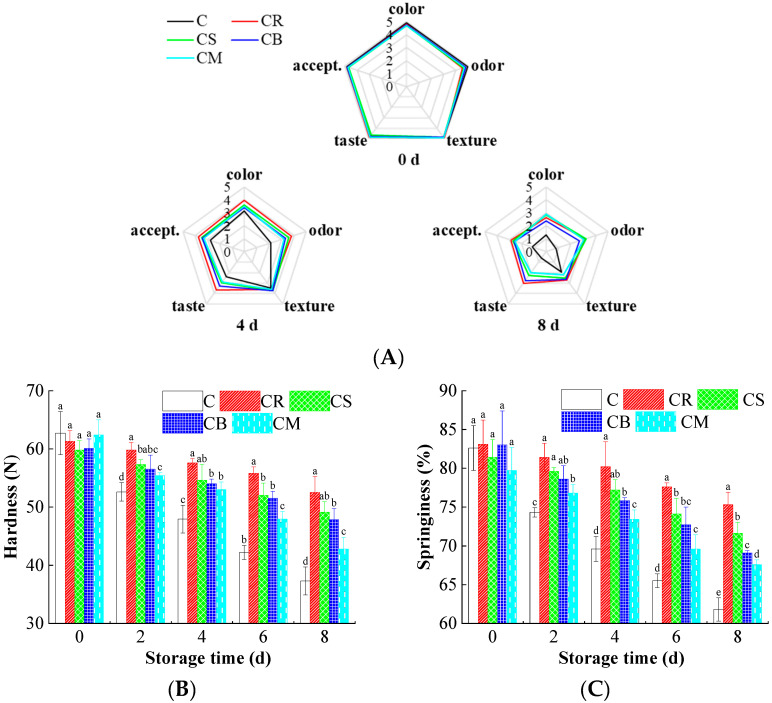
The effects of the treatments using Citrus peels on the sensory decline of beef. (**A**) Sensory scores; (**B**) Hardness; (**C**) Springiness. Different letters (a~e) indicate the significant difference (*p* < 0.05) between each treatment.

Figure 1A illustrates the sensory evaluation of beef treated with Citrus peels over the storage period. While all samples exhibited a gradual decline in sensory quality, the treated beef demonstrated significantly better preservation of color, texture, and overall acceptability, highlighting the extract’s protective effects [43,44]. This improvement can be attributed to the bioactive compounds in Citrus peel-flavonoids, terpenoids, and alkaloids-which possess strong antioxidant and antimicrobial properties [45]. These compounds collectively mitigate oxidative stress and microbial spoilage, thereby slowing the deterioration and extending beef palatability. Specifically, the preservation of a bright, red color can be linked to the antioxidant capacity of flavonoids and terpenoids (e.g., limonene) in reducing the oxidation of oxymyoglobin to metmyoglobin, while the maintenance of pleasant odor correlates with the suppression of volatile off-flavor compounds generated from lipid oxidation and microbial metabolism [46,47].

Further analysis of texture parameters, including hardness (Figure 1B) and springiness (Figure 1C), revealed a significant decline in control samples over time, primarily due to moisture loss, protein degradation, and lipid oxidation. However, the decline was markedly slower in the treated samples, indicating a pronounced ability to retain muscle integrity. This effect may stem from its ability to inhibit proteolytic enzyme activity and stabilize muscle fiber structure, thereby preserving the mechanical properties of beef during storage [15]. The superior performance of CR treatment across all sensory and textural parameters consistently aligns with its highest content of limonene and strongest overall bioactivity, establishing a clear structure-function relationship between the chemical composition of the peel and its efficacy in quality preservation [37].

### 3.4. Microbial and Chemical Degradation in the Beef

**Figure 2:**
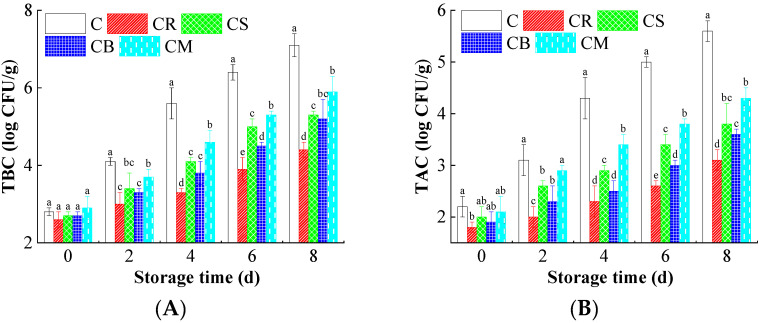

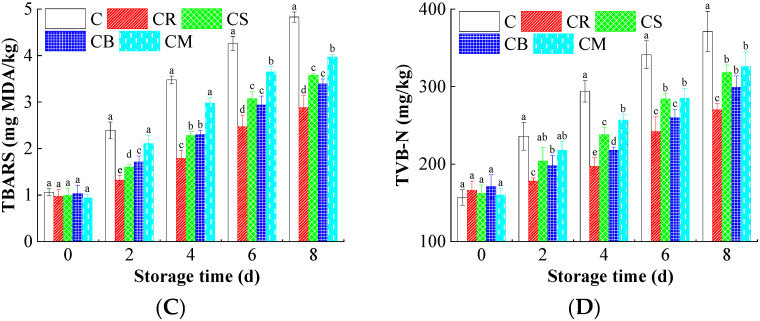
The effects of the treatments using Citrus peels on the microbial infection, lipid oxidation, and protein degradation of beef. (**A**) Total bacterial count (TBC); (**B**) Total aerobic count (TAC); (**C**) Thiobarbituric acid reactive substances (TBARS); and (**D**) Total volatile basic nitrogen (TVB-N). Different letters (a~e) indicate the significant difference (*p* < 0.05) between each treatment.

Microbial proliferation is a primary determinant of meat safety and shelf life. As shown in Figure 2A,B, the control group exhibited the most rapid microbial growth, with TBC and TAC reaching final values of 7.1 ± 0.3 and 5.6 ± 0.2 log CFU/g, respectively, by day 8. In marked contrast, beef treated with CR peel extract maintained significantly (*p* < 0.05) lower microbial loads, with final TBC and TAC values of 4.4 ± 0.2 and 3.1 ± 0.2 log CFU/g, representing significant reduction compared to the control. The inhibitory effect of Citrus peels can be attributed to the synergistic action of its flavonoids (e.g., hesperidin, naringin) and terpenoids (e.g., limonene, γ-terpinene) [2,48,49]. These compounds exert broad-spectrum antimicrobial effects through multiple, often synergistic, mechanisms [22,50]: Firstly, membrane disruption serves as a primary mode of action: lipophilic terpenoids, such as limonene, can integrate into and destabilize the bacterial lipid bilayer. This interaction increases membrane fluidity and permeability, culminating in the leakage of vital intracellular ions and constituents, ultimately leading to cell lysis. Secondly, enzyme inhibition contributes significantly to antimicrobial efficacy. Flavonoids can bind to and inactivate key bacterial enzymes, particularly those involved in ATP synthesis and cell wall formation, thereby crippling essential metabolic pathways and compromising microbial viability. Furthermore, the induction of intracellular oxidative stress represents another critical mechanism. Certain polyphenolic components can provoke the accumulation of reactive oxygen species (ROS) within bacterial cells, overwhelming their antioxidant defense systems and causing oxidative damage to proteins, lipids, and DNA, which disrupts cellular functions and inhibits proliferation [51,52]. The combined impact of these mechanisms effectively slows microbial spoilage, thereby enhancing the beef safety and shelf-life stability of beef.

Lipid and protein oxidation significantly contribute to meat deterioration [15]. Figure 2C presents changes in TBARS, a key marker of lipid oxidation. Over time, TBARS values increased in all samples, but the rise was significantly lower in Citrus peel-treated beef. By the end of storage, TBARS levels in the control group reached 4.83 mg MDA/kg, whereas treated samples exhibited considerably lower values (ranging from 2.88 mg/kg to 3.97 mg/kg), demonstrating the extract’s effectiveness in inhibiting lipid peroxidation.

The antioxidant efficacy is primarily mediated by flavonoids, which act as potent free radical scavengers to neutralize ROS such as hydroxyl radicals (•OH), superoxide anions (O_2_•^−^), and singlet oxygen (^1^O_2_), thereby preventing oxidative degradation of biomolecules [30]. The underlying mechanisms involve (1) hydrogen atom transfer (HAT), where flavonoids donate hydrogen atoms to terminate free radical chain reactions, stabilizing them and halting chain reactions of oxidation; (2) metal ion chelation, whereby specific flavonoids sequester pro-oxidant transition metals like Fe^2+^ and Cu^2+^ to inhibit metal-catalyzed oxidation; and (3) phenoxy radical stabilization, facilitated by electron delocalization within the flavonoid structure, which prevents propagation of oxidative damage. The superior antioxidant performance of CR extract likely stems from its high flavonoid content complemented by synergistic interactions with limonene, collectively enhancing the oxidative stability of the beef system.

Similarly, Figure 2D shows changes in TVB-N, an indicator of protein degradation. The control group’s TVB-N levels surged from 157 mg/kg to 371 mg/kg, reflecting extensive proteolysis. In contrast, the treated samples exhibited significantly lower TVB-N levels (270–326 mg/kg), indicating that Citrus peels effectively slow protein degradation. Protein degradation is primarily driven by microbial and endogenous enzymatic activity, leading to the breakdown of amino acids into volatile nitrogenous compounds. Citrus peel extracts mitigate protein degradation through a multi-target mechanism: primarily by inhibiting proteolytic bacteria to suppress enzyme production. Concurrently, their antioxidant constituents reduce ROS-induced protein oxidation, thereby preserving structural integrity. Additionally, specific bioactive compounds may directly interact with muscle proteins to diminish their susceptibility to enzymatic breakdown [10,22].

### 3.5. Microstructure Variations in the Beef

**Figure 3:**
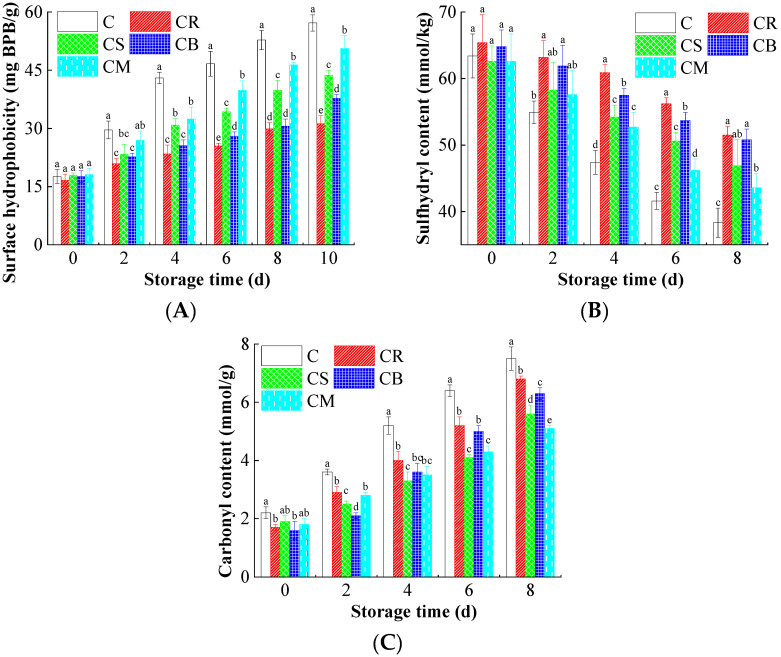
The effects of the treatments using Citrus peels on the variations in cellular microstructure of beef. (**A**) Surface hydrophobicity; (**B**) Sulfhydryl content; and (**C**) Carbonyl content. Different letters (a~e) indicate the significant difference (*p* < 0.05) between each treatment.

The integrity of beef microstructure is fundamentally linked to its textural quality and water-holding capacity during storage [15]. Figure 3A–C revealed significant alterations in key microstructural indicators under oxidative and microbial stress, which were effectively mitigated by Citrus peel treatment.

Hydrophobicity increased significantly in control samples over storage, indicating protein unfolding and exposure of hydrophobic residues (Figure 3A) [18]. This denaturation impairs protein functionality, leading to reduced water binding and textural deterioration (e.g., increased toughness or dryness). Citrus peel treatments, particularly CR, markedly suppressed this rise. This stabilization is attributed to the antioxidant flavonoids (e.g., hesperidin) scavenging reactive oxygen species (ROS), thereby preventing protein unfolding and maintaining native conformation.

Free thiol groups (-SH) are critical for protein tertiary structure via disulfide bonds [15]. Oxidative stress promotes disulfide bond formation (-S-S-), decreasing free SH content and destabilizing protein networks. In this study, control beef exhibited a pronounced decline in SH groups (Figure 3B). In contrast, Citrus peel treated samples, especially CR, preserved significantly higher SH levels. This protection stems directly from the ROS-scavenging capacity of peel constituents (e.g., limonene, flavonoids), preventing oxidation of cysteine residues and preserving protein structural integrity crucial for texture and juiciness.

Carbonyl formation results from direct oxidative attack on amino acid side chains (e.g., Lys, Arg, Pro), serving as a primary marker of protein oxidation [22]. As shown in Figure 3C, control samples showed a substantial accumulation of carbonyls. Citrus peel treatments significantly inhibited this accumulation, with CR again demonstrating the strongest effect. This inhibition is mechanistically linked to the peel’s antioxidants neutralizing ROS before they can react with amino acid residues. Limiting carbonyl formation prevents protein fragmentation, aggregation, and loss of functionality, thereby preserving muscle fiber structure and associated textural properties like hardness and elasticity.

Collectively, these microstructural alterations reflect progressive chemical oxidation and protein denaturation, which are key drivers of quality deterioration in beef. Citrus peel extract counteracts this deterioration primarily via potent ROS scavenging by its bioactive constituents, including flavonoids and terpenoids. By neutralizing free radicals derived from lipid oxidation and microbial activity, the extract effectively shields muscle proteins from oxidative damage. This structural preservation maintains water-holding capacity, stabilizes the myofibrillar network, and suppresses the formation of off-flavor compounds. Thus, the traditional practice of “citrus peel beef” is scientifically grounded, as it sustains the microstructural integrity essential for sensory quality and shelf-life extension.

### 3.6. Biogenic Amines Accumulation in the Beef

**Figure 4:**
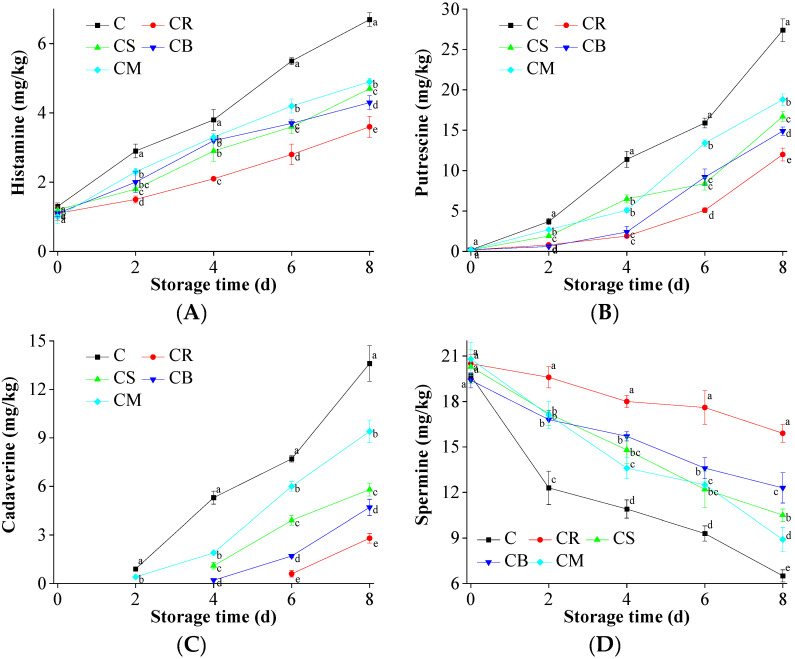

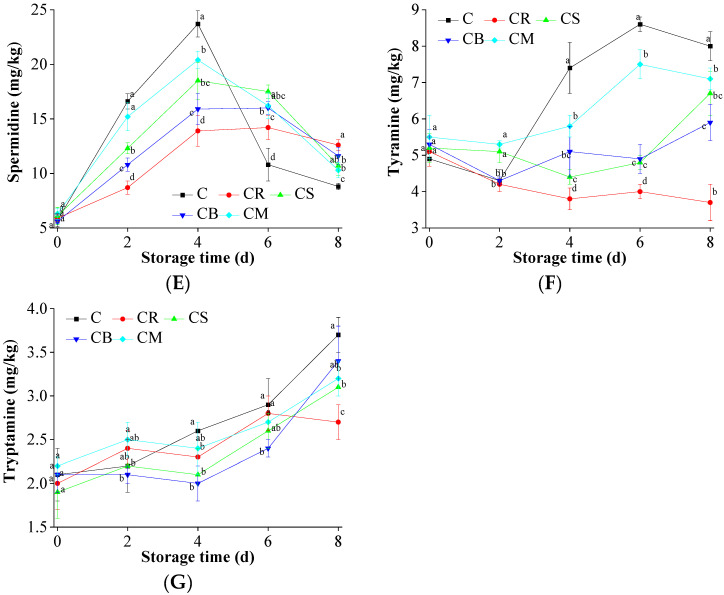
The effects of the treatments using Citrus peels on the accumulation of biogenic amines in the preserved beef. (**A**) Histamine; (**B**) Putrescine; (**C**) Cadaverine; (**D**) Spermine; (**E**) Spermidine; (**F**) Tyramine; and (**G**) Tryptamine. Means in same column with different letters are significantly different (*p* < 0.05).

The accumulation of biogenic amines, including putrescine, cadaverine, and histamine, is a key biochemical marker of microbial spoilage and protein degradation in stored beef [18]. Figure 4A–G illustrates the temporal changes in biogenic amine content during storage. In the control group, putrescine levels escalated dramatically from 0.2 mg/kg to 27.4 mg/kg over 8 days, whereas beef treated with Citrus peels exhibited significantly lower putrescine accumulation, with final levels ranging from 12 mg/kg to 18.8 mg/kg. Similar trends were observed for cadaverine and histamine, reinforcing the inhibitory effects of Citrus peel on microbial metabolic pathways responsible for amine formation.

The biosynthesis of biogenic amines is enzymatically driven, primarily by spoilage-associated bacteria, including *Lactobacillus* and *Enterobacter* species, under anaerobic conditions [15]. Ornithine decarboxylase and spermidine synthase catalyze the conversion of putrescine into spermidine and spermine, which are involved in bacterial stress responses and metabolic regulation. The antimicrobial properties of Citrus peel components, particularly terpenoids (e.g., limonene, γ-terpinene) and flavonoids (e.g., hesperidin, naringin), disrupt these enzymatic pathways by inhibiting microbial proliferation and suppressing decarboxylase activity [18,53]. Specifically, terpenoids integrate into bacterial lipid membranes, destabilizing their structure and increasing permeability, which leads to loss of intracellular homeostasis and ultimately cell death. Flavonoids, on the other hand, exhibit chelating activity, potentially interfering with the metal-dependent enzymatic functions necessary for biogenic amine synthesis [22].

Histamine formation, catalyzed by histidine decarboxylase, showed a more moderate reduction in Citrus peel-treated samples [14]. This suggests that some Gram-negative bacteria, particularly Enterobacteriaceae, may exhibit higher resistance to phenolic and terpenoid-based antimicrobials due to their outer membrane barrier and efflux pump mechanisms [18]. However, despite this partial resistance, the overall suppression of biogenic amine formation indicates that Citrus peels effectively inhibit microbial spoilage pathways, delaying protein degradation and extending beef shelf life.

In summary, Citrus peel treatment enhances beef storage stability by inhibiting microbial activity, suppressing oxidative degradation, and modulating enzymatic pathways involved in biogenic amine formation. Through a multifaceted mechanism—including free radical scavenging, bacterial membrane disruption, and enzymatic inhibition—the bioactive constituents of Citrus peel effectively preserve sensory attributes, maintain nutritional integrity, and improve food safety. These findings underscore the potential of citrus-derived natural preservatives as a sustainable strategy to mitigate spoilage and extend the shelf life of perishable meat products.

### 3.7. Correlation Analysis and Mechanistic Insights

**Figure 5:**
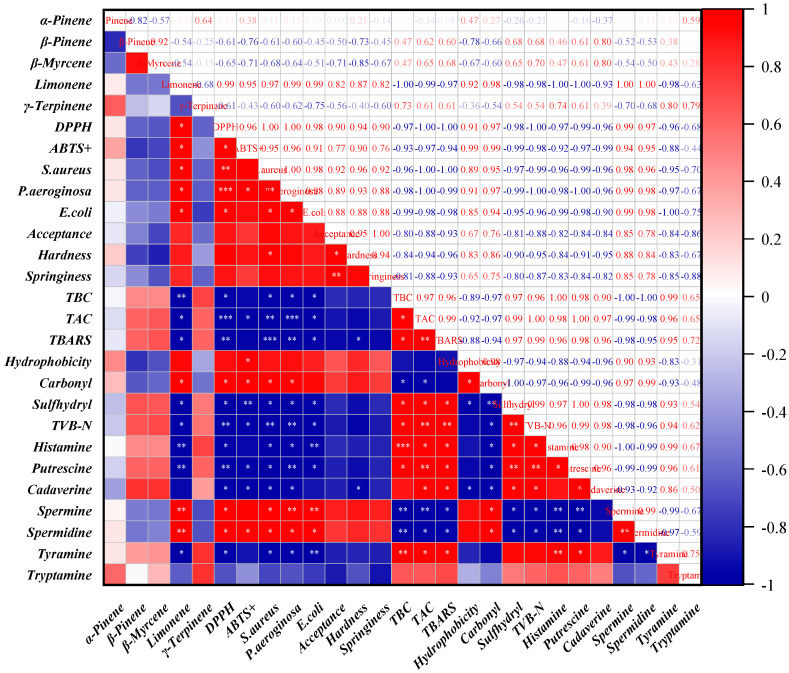
Pearson correlation coefficients among various parameters during the storage of Citrus peel beef. The symbol *, **, and *** indicate the significant level of *p* ≤ 0.05, *p* ≤ 0.01, and *p* ≤ 0.001, respectively.

The correlation analysis provided critical insights into the relationships between key Citrus peel components, their antioxidant and antibacterial properties, and the quality evolution of beef during storage (Figure 5). As the predominant compound in Citrus peels, limonene exhibited a strong positive correlation (*p* < 0.05) with DPPH and ABTS^+^ radical scavenging capacity, confirming its major contribution to the antioxidant potential of Citrus peels. The antioxidant mechanism of limonene is primarily attributed to its ability to scavenge free radicals through hydrogen atom transfer, thereby mitigating oxidative stress and delaying lipid peroxidation.

Beyond its antioxidant role, limonene also displayed a significant correlation (*p* < 0.05) with the inhibition zones of *S. aureus*, *P. aeruginosa*, and *E. coli*, underscoring its crucial role in antibacterial activity. The antimicrobial mechanism is likely associated with its high lipophilicity, which facilitates membrane integration, disrupts lipid bilayer stability, and compromises bacterial homeostasis. This disruption leads to increased membrane permeability, leakage of intracellular components, and eventual cell lysis, thereby inhibiting bacterial proliferation [15].

The antioxidant and antibacterial properties of Citrus peel extracts were strongly correlated (*p* < 0.05) with multiple quality parameters of beef during storage. On one hand, the free radical scavenging activity of Citrus peel components significantly suppressed lipid peroxidation and protein degradation, as reflected in reduced TBARS and TVB-N values. On the other hand, the antibacterial effects of Citrus peel extracts effectively restrained microbial growth, resulting in significantly lower TBC and TAC levels (*p* < 0.05) in beef samples. These combined effects contributed to the preservation of beef sensory attributes, including improved textural integrity (higher elasticity and hardness) and the prevention of secondary oxidative products responsible for off-flavors [22].

Furthermore, biogenic amine accumulation was significantly negatively correlated (*p* < 0.05) with the antioxidant and antibacterial properties of Citrus peel extracts. The formation of biogenic amines requires both proteolytic degradation of proteins to release amino acid precursors and bacterial enzymatic activity to catalyze their decarboxylation. The suppression of spoilage-related bacteria, particularly *Enterobacteriaceae* and *Pseudomonas* spp., the primary producers of biogenic amines, directly contributed to lower levels of histamine, putrescine, tyramine, and other amines in stored beef [15]. This aligns with previous studies highlighting the direct link between microbial control and reduced amine accumulation in meat products [15].

In summary, the bioactive components of Citrus peel, particularly limonene, play a pivotal role in enhancing beef stability and safety during storage through their potent antioxidant and antibacterial activities. By mitigating oxidative deterioration, inhibiting microbial proliferation, and reducing biogenic amine formation, these natural compounds offer a promising alternative to synthetic preservatives in meat preservation. These findings provide a mechanistic foundation for the application of Citrus peels as an effective natural preservation strategy, supporting the development of safer and more sustainable meat storage solutions.

## 4. Conclusions

This study highlights the promising potential of Citrus peel beef as a model for applying traditional preservation techniques in modern food science. The results demonstrate that Citrus peels significantly enhance beef quality by reducing lipid oxidation (TBARS) levels, inhibiting protein degradation (TVB-N) and maintaining microstructure (surface hydrophobicity, sulfhydryl and carbonyl content). The extract also effectively suppresses microbial growth, extending the shelf life of beef, while preventing the formation of biogenic amines, especially putrescine, a key spoilage indicator. These findings validate the preservative efficacy of Citrus peel, offering a natural solution to improve both the safety and sensory attributes of beef. The identified efficacy of limonene-rich peel extracts, particularly from Citrus reticulata, suggests a strong potential for developing natural preservative formulations or active packaging systems for the meat industry. This research bridges the gap between ancient culinary practices, such as the use of Citrus peel in Citrus peel beef, and modern scientific perspectives, offering a scientifically grounded approach to solving contemporary food preservation challenges. By integrating traditional methods with modern techniques, this study presents a sustainable and effective strategy for enhancing food safety and extending shelf life in the meat industry.

## Figures and Tables

**Table 1:** Main effects composition in the Citrus peels.

RI	Components	%RA				Reported Bio-Activity
		CR	CS	CB	CM	
937	α-Pinene	3.9 ± 0.2 a	4.7 ± 1.2 a	5.4 ± 0.7 a	3.6 ± 1.1 a	Antimicrobial [32], antioxidant [33]
974	Sabinene	1.6 ± 0.3 b	3.5 ± 0.6 a	1.1 ± 0.3 b	0.9 ± 0.5 b	Antimicrobial [34], antioxidant [35]
979	β-Pinene	2.4 ± 0.7 b	1.4 ± 0.4 c	0.6 ± 0.1 d	7.4 ± 1.3 a	Antimicrobial [22], antioxidant [36]
987	β-Myrcene	4.2 ± 1.1 a	3.8 ± 1.1 a	4.4 ± 0.6 a	5.9 ± 0.7 a	Antioxidant [10]
1030	Limonene	77.1 ± 2.6 a	63.5 ± 4.1 b	68.9 ± 4.5 b	59.6 ± 5.2 b	Antimicrobial [37], Antioxidant [31]
1059	γ-Terpinene	4.7 ± 0.3 c	9.5 ± 0.6 a	8.7 ± 0.8 ab	7.4 ± 1.0 b	Antimicrobial, antioxidant [38]
1185	α-Terpinolene	0.9 ± 0.6 a	0.8 ± 0.2 b	1.4 ± 0.3 a	1.9 ± 0.7 a	Antimicrobial [39]
1094	Linalool	1.4 ± 0.5 ab	2.8 ± 1.2 a	2.1 ± 0.7 a	0.9 ± 0.1 b	Antimicrobial, antioxidant [10]
1005	Octanal	1.1 ± 0.7 b	3.0 ± 0.8 a	3.6 ± 0.3 a	1.8 ± 0.6 ab	Antimicrobial, antioxidant [10]
1203	Decanal	0.9 ± 0.1 b	1.2 ± 0.3 b	0.7 ± 0.2 b	3.4 ± 1.4 a	Antimicrobial, antioxidant [40]
	Total	98.2 ± 1.4	94.2 ± 4.3	96.9 ± 2.4	92.8 ± 5.0	

RI: Retention index relative to n-alkanes on HP-5 MS capillary column. %RA: Relative area (peak area relative to the total peak area). Different letters (a~d) indicate the significant difference (*p* < 0.05) between each treatment.

**Table 2:** Antioxidant and antimicrobial activities of the Citrus peels.

Activities		CR	CS	CB	CM
Antioxidant activity (%)	DPPH	60.8 ± 3.1 a	49.6 ± 1.8 b	52.1 ± 2.0 b	43.2 ± 2.7 b
	ABTS^+^	66.0 ± 3.6 a	54.9 ± 2.2 b	61.5 ± 4.9 ab	46.4 ± 1.8 c
Antimicrobial zone (mm)	*S. aureus*	11.6 ± 0.8 a	9.5 ± 0.3 b	9.8 ± 0.5 b	8.1 ± 0.4 c
	*L. monocytogenes*	13.4 ± 0.7 a	10.8 ± 0.3 b	9.9 ± 0.8 bc	8.7 ± 0.6 c
	*P. aeroginosa*	8.6 ± 0.6 a	6.7 ± 0.2 b	7.2 ± 0.6 b	5.7 ± 0.5 c
	*E. coli*	11.0 ± 0.2 a	7.9 ± 0.5 bc	8.6 ± 0.3 b	7.1 ± 0.4 c
	*S. typhimurium*	9.4 ± 0.5 a	7.1 ± 0.5 bc	7.6 ± 0.4 b	6.5 ± 0.3 c
MIC (mg/mL)	*S. aureus*	4.8 ± 0.2 c	5.7 ± 0.2 b	5.9 ± 0.4 b	6.9 ± 0.3 a
	*L. monocytogenes*	4.1 ± 0.3 d	4.9 ± 0.4 c	5.7 ± 0.1 b	6.3 ± 0.3 a
	*P. aeroginosa*	7.9 ± 0.5 c	9.5 ± 0.5 b	8.9 ± 0.6 bc	12.1 ± 0.8 a
	*E. coli*	5.1 ± 0.4 c	5.5 ± 0.2 c	6.4 ± 0.3 b	8.6 ± 0.6 a
	*S. typhimurium*	8.3 ± 0.6 c	9.7 ± 0.5 b	9.0 ± 0.5 bc	11.4 ± 0.8 a

Different letters (a~d) indicate the significant difference (*p* < 0.05) between each treatment.

## Data Availability

The original contributions presented in this study are included in the article. Further inquiries can be directed to the corresponding authors.
